# MRI Evaluation of Traumatic and Non-traumatic Pathologies of the Ankle Joint and Hindfoot: A Single-Center Observational Study

**DOI:** 10.7759/cureus.67103

**Published:** 2024-08-18

**Authors:** Anuradha Kelkar, Ojasvi Sharma, Saksham Jain, Sayali Paidlewar, Ankita Pandey, Sravya Julakanti, Akhil Varma

**Affiliations:** 1 Department of Radiology, Dr. D. Y. Patil Medical College, Hospital and Research Centre, Dr. D. Y. Patil Vidyapeeth (Deemed to be University), Pune, IND

**Keywords:** magnetic resonance imaging (mri), ankle joint-foot, imaging findings, hind foot, ankle joint

## Abstract

Background

Pathologies affecting the ankle joint and hindfoot can present with a variety of clinical symptoms and etiologies, necessitating accurate diagnostic tools for effective management. Magnetic resonance imaging (MRI) is a valuable imaging modality for assessing these pathologies, providing detailed visualization of bone, joint, tendon, and other soft tissue abnormalities.

Objectives

To evaluate MRI findings in a diverse cohort of 105 participants with pathologies affecting the ankle joint and hindfoot, focusing on the prevalence and types of bone, joint, tendon, and soft tissue abnormalities.

Materials and methods

A single-center observational descriptive study was conducted at Dr. D. Y. Patil Medical College and Hospital and Research Centre, Pune, India, over a period from August 2022 to July 2024, involving 105 participants (54.3% male, 45.7% female) with a mean age of 39.04 years. MRI scans were analyzed to assess the prevalence of bone, joint, tendon, and soft tissue pathologies. Clinical profiles, symptom duration, and etiological classifications were documented.

Results

Analysis of the results obtained from 105 (N = 105) study participants revealed that pain (94.3%, or 99 cases) was the most common symptom, followed by restricted movement (86.7%, or 91 cases), trauma history (75.2%, or 79 cases), and swelling (73.3%, or 77 cases). Traumatic causes (76.2%, or 80 cases) predominated, while inflammatory (48.3%, or 14 cases) and infective (34.5%, or 10 cases) causes were also significant. MRI findings showed marrow edema in 41.9%, or 44 cases, subchondral cysts in 22.9% (24 cases), fractures in 17.1% (18 cases), and erosions in 10.5% of participants (11 cases). Joint involvement was most frequent in the tibiotalar (76.2%, or 80 cases) and subtalar joints (58.1%, or 61 cases). Tendon pathologies included peritendonitis (55.2%, or 58 cases) and tendinosis (23.8%, or 25 cases), with the Achilles tendon being the most frequently affected (39%, or 41 cases). Ligament injuries were predominantly sprains (46.7%, or 49 cases), with less frequent partial (18.1%, or 19 cases) and complete tears (7.6%, or eight cases). Soft tissue findings included subcutaneous edema (76.2%, or 80 cases) and bursitis (24.8%, or 26 cases). Among the study participants who presented with non-traumatic pathologies, inflammatory pathologies (48.3%, or 14 cases) were the most common, followed by infective (34.5%, or 10 cases) and neoplastic (17.2%, or five cases) pathologies.

Conclusion

MRI effectively identifies a wide range of pathologies in the ankle and hindfoot, with marrow edema, joint effusion, and tendon pathologies being prevalent. The study underscores the utility of MRI in diagnosing and assessing various conditions in the ankle joint complex and highlights the need for accurate imaging to guide treatment decisions. Future research should focus on correlating MRI findings with clinical outcomes to enhance diagnostic accuracy and management strategies.

## Introduction

Magnetic resonance imaging (MRI) is highly effective in promptly identifying and evaluating various bone abnormalities, including stress fractures, bone contusions, osteochondral fractures, transient bone marrow edema, and osteonecrosis [1].

The ankle joint and hindfoot are crucial anatomical regions subjected to significant mechanical stress, often leading to a variety of traumatic and non-traumatic pathologies. These areas play a pivotal role in mobility, bearing weight, and providing balance, making them susceptible to injuries and degenerative conditions [2]. MRI has emerged as an invaluable tool in the diagnostic evaluation of these pathologies, due to its superior soft tissue contrast and multiplanar imaging capabilities [3].

Traumatic injuries to the ankle joint and hindfoot are common, especially among athletes and individuals engaged in physical activities. These injuries can range from ligament sprains and tendon tears to fractures and dislocations. Accurate and timely diagnosis is essential to guide appropriate management and rehabilitation, preventing long-term complications and ensuring optimal functional recovery [4]. MRI's ability to visualize soft tissues, cartilage, and bone marrow in detail makes it the modality of choice for detecting subtle injuries that may not be apparent on conventional radiographs [3].

Non-traumatic pathologies of the ankle and hindfoot, such as osteoarthritis, rheumatoid arthritis, and congenital deformities, also pose significant challenges in clinical practice. These conditions often lead to chronic pain, stiffness, and functional impairment, impacting the quality of life [5]. MRI is particularly useful in identifying early degenerative changes, inflammatory processes, and other subtle abnormalities, allowing for early intervention and improved patient outcomes [6].

The anatomy of the ankle joint and hindfoot is complex, comprising bones, ligaments, tendons, and other soft tissue structures. A thorough understanding of this anatomy is essential for accurate interpretation of MRI findings [2]. The ankle joint includes the tibiotalar, subtalar, and transverse tarsal joints, each with specific biomechanical functions. Ligamentous structures, such as the lateral collateral ligaments, medial collateral ligaments, and syndesmotic ligaments, provide stability, while tendons, including the Achilles tendon, facilitate movement [7].

The biomechanics of the ankle joint further complicate the diagnostic evaluation of pathologies [8]. The ankle joint permits dorsiflexion and plantar flexion, while the subtalar joint allows for inversion and eversion movements. Stability is maintained through the intricate interplay of ligamentous and tendinous structures, and any disruption can lead to significant functional impairment. Understanding these biomechanical principles is crucial for identifying the underlying causes of pain and dysfunction in the ankle and hindfoot [9]. The medial collateral ligament, or deltoid ligament complex, is the principal ankle ligament responsible for stabilizing the ankle when it is subjected to axial loading. It has five bands - the anterior and posterior tibiotalar, tibiospring, tibiocalcaneal, and tibionavicular ligaments. Injury to this structure is quite rare and occurs in just 5% of cases of ankle sprains [10].

Ankle sprains are anatomically classified according to the number of ligaments that are affected. A complete or partial tear of the anterior talofibular ligament (ATFL) characterizes a first-degree sprain. A partial or full tear of the ATFL, as well as the calcaneofibular ligament (CFL), characterizes a second-degree sprain. CFL, ATFL, and posterior talofibular ligament (PTFL) are all involved in third-degree sprains [11].

Among the prevalent infectious pathologies, MRI is the preferred modality for early detection of joint tuberculosis. Musculoskeletal tuberculosis affects 1-3% of patients with TB, with axial skeletal involvement being more frequent. Peripheral arthritis is reported in approximately 60% of cases, while osteomyelitis occurs in about 38% of cases [12]. Tuberculous osteomyelitis is more common in the bones of the extremities. Changes in the bone marrow are visualized as regions of decreased and increased signal intensity on T1-weighted and T2-weighted images, respectively, and exhibit notable enhancement after contrast administration. Deep soft tissue alterations, such as sinus tracts or abscess formation, are more effectively depicted on gadolinium-enhanced images [13].

The literature reveals extensive use of MRI in diagnosing ankle joint and hindfoot pathologies. Numerous studies have demonstrated MRI's superiority in detecting both acute injuries and chronic conditions, often leading to changes in clinical management. By compiling and analyzing existing research, this paper underscores the importance of MRI in the diagnostic process and its impact on treatment outcomes. Through a comprehensive review of traumatic and non-traumatic pathologies, this study aims to highlight the indispensable role of MRI in modern clinical practice, ultimately contributing to better patient care and outcomes [3,6].

The objective of this study is to evaluate MRI findings in a diverse cohort of 105 participants with pathologies affecting the ankle joint and hindfoot, with a focus on the prevalence and classification of bone, joint, tendon, and soft tissue abnormalities on MRI.

## Materials and methods

Study setting and design

A single-center observational descriptive study was conducted at Dr. D. Y. Patil Medical College, Hospital and Research Centre, Pune, India, over a period from August 2022 to July 2024.

Inclusion and exclusion criteria

The study included 105 cases. Participants were selected based on specific inclusion criteria, which encompassed individuals of all ages, both men and women, presenting with pain in the ankle joint and hindfoot, whether due to trauma or without a history of trauma. Conditions such as fractures, dislocations, tumorous conditions, infections, amputations, and inflammatory arthritis were included. Exclusion criteria involved patients with orthopedic hardware, cardiac pacemakers, metallic foreign bodies, cochlear implants, an inability to remain still during the MRI scan, claustrophobia, and those with a history of allergic reactions to contrast agents.

Technique

MRI scans were performed using a Siemens Magnetom VIDA 3 Tesla MRI machine (Siemens Healthineers, Erlangen, Germany). Patients were positioned supine, with the medial malleolus centered in the coil. The foot was relaxed in approximately 10 to 20 degrees of plantar flexion and 10 to 30 degrees of external rotation. An extremity surface coil was used to enhance spatial resolution. Scans were conducted in axial, sagittal, and coronal planes. Contrast studies were performed as needed.

Technical protocols

The MRI sequences employed are shown in Table 1.

**Table 1:** MRI sequences employed WI: Weighted imaging; FSE: Fast spin echo; STIR: Sagittal short tau inversion recovery; PDFS: Proton density fat suppression; TR: Repetition time; TE: Time to echo; FoV: Field of view; GRE: Gradient echo; FS: Fat suppression

Sequence	TR (ms)	TE (ms)	Slice thickness (mm)	FoV (mm)	Flip angle (°)	Bandwidth (Hz/pixel)
Axial T1 WI FSE	663	8.9	3	250	150	191
Coronal T1 WI FSE	600	8.9	3	250	120	191
Sagittal T1 WI FSE	663	8.9	3	250	120	191
STIR	2750	30	3	250	150	190
Coronal T2 WI FSE	3500	80	3	250	50	196
Axial PDFS	3005	36	3	150	150	182
Sagittal PDFS	3000	35	3	160	150	182
T2 GRE	480	15	3	250	20	100
Axial T1 FS WI	663	8.9	3	250	150	191
Coronal T1 FS WI	600	8.9	3	250	120	191
Sagittal T1 FS WI	663	8.9	3	250	120	191

Statistical analysis

Data were collected and recorded over a two-year period and subsequently analyzed using Microsoft Excel (Microsoft® Corp., Redmond, WA, USA) and IBM SPSS Statistics for Windows, Version 26.0 (Released 2019; IBM Corp., Armonk, NY, USA). Categorical variables were summarized using frequencies and proportions, while continuous variables were analyzed with means and standard deviations.

## Results

The study comprised 105 participants, of whom 57 were male (54.3%) and 48 were female (45.7%). The age distribution was diverse, with the majority of participants falling within the 21-30 age group (28.6%), followed by the 31-40 age group (23.8%) (Table 2). The mean age of the participants was 39.04 years, with a standard deviation of 18.77 years.

**Table 2:** Distribution of study participants on the basis of age

Age (years)	Frequency (N = 105)	Percentage
<20	12	11.4%
21-30	30	28.6%
31-40	25	23.8%
41-50	6	5.7%
51-60	12	11.4%
61-70	12	11.4%
>70	8	7.6%
Total	105	100.0

The clinical profile of the study participants showed that pain was the most common symptom, reported by 94.3% (99 cases) of the participants. Other prevalent symptoms included restricted movement (86.7%, or 91 cases), history of trauma (75.2%, or 79 cases), and swelling (73.3%, or 77 cases). Less common symptoms included twisting injuries (34.3%, or 36 cases), fall from height (19%, or 20 cases), and discharging sinus (10.5%, or 11 cases) (Table 3). The duration of symptoms varied, with 31.4% (33 participants) experiencing acute symptoms (less than or equal to four weeks) and 68.6% (72 participants) experiencing chronic symptoms (more than four weeks).

**Table 3:** Clinical profile of study participants * Multiple responses for each study participant allowed

Clinical profile*	Frequency (N = 105)	Percentage
Pain	99	94.3%
Swelling	77	73.3%
History of trauma	79	75.2%
Twisting injury	36	34.3%
Discharging sinus	11	10.5%
Fall from height	20	19.0%
Restricted movement	91	86.7%
Ulceration	9	8.6%
Discoloration	6	5.7%
History of recent TB or TB contact	1	1.0%
Diabetes	10	9.5%
History of relevant surgery	4	3.8%
Fever	9	8.6%

The etiological classification revealed that traumatic causes were predominant, accounting for 76.2% of cases. Among the non-traumatic causes, inflammatory pathologies were the most common (48.3%, or 14 cases), followed by infective causes (34.5%, or 10 cases) and neoplastic causes (17.2%, or five cases). Regarding non-traumatic pathologies, the most frequently observed conditions were inflammatory (48.3%, or 14 cases) and infective (34.5%, or 10 cases), with neoplastic causes being less common (17.2%, or five cases). This data emphasizes the importance of differentiating between various non-traumatic conditions to ensure appropriate diagnosis and treatment (Table 4).

**Table 4:** Distribution of non-traumatic pathologies among study participants * Includes infections and inflammatory pathologies following a history of trauma

Non-traumatic cause of lesion	Frequency (N = 29)	Percentage
Infective*	10	34.5%
Inflammatory*	14	48.3%
Neoplastic	5	17.2%

The MRI findings in this study revealed significant involvement of various bones in the ankle joint and hindfoot, underscoring the importance of MRI in identifying osseous abnormalities. The talus was the most frequently involved bone, with abnormalities detected in 41% of participants, highlighting its central role in ankle joint mechanics and the common occurrence of talar pathologies. The calcaneum, or heel bone, was the second most commonly affected, observed in 29.5%, or 31 cases, indicating the prevalence of calcaneal injuries that often result in heel pain and mobility issues. The lower tibia and lower fibula were also notably affected, found in 21% (22 cases) and 15.2% (16 cases) of participants, respectively, underscoring their susceptibility to both traumatic and non-traumatic pathologies. Less commonly involved bones included the navicular (4.8%, or five cases), cuboid (5.7%, or six cases), and cuneiform (6.7%, or seven cases), each playing critical roles in foot stability and function. Additionally, accessory ossicles were identified in 3.8%, or four cases, which, although often asymptomatic, can sometimes cause pain and require precise diagnosis (Table 5).

**Table 5:** Bone involvement among study participants * Multiple responses for each study participant allowed

Bone*	Frequency (N = 105)	Percentage
Lower tibia	22	21%
Lower fibula	16	15.2%
Talus	41	39%
Calcaneum	31	29.5%
Navicular	5	4.8%
Cuboid	6	5.7%
Accessory ossicles	4	3.8%
Cuneiform	7	6.7%

The most common MRI finding in patients with bone involvement was marrow edema, identified in 44 cases, which represents 41.9% of the study population. Subchondral or intraosseous cysts were found in 24 cases, or 22.9%. Fractures and erosions were also relatively common, observed in 18 (17.1%) and 11 (10.5%) cases, respectively. Contusions were noted in nine cases (8.6%), while serpiginous areas of altered signal intensity and subchondral compression were less frequent, appearing in two cases (1.9%) and one case (1%), respectively. Other findings, such as the Stieda process, osteophytes, and sclerotic lesions, were observed in eight cases (7.6%), eight cases (7.6%), and one case (1%), respectively. Neoplastic lesions, periosteal reactions/periostitis, sequestrum, abscess, and apophysitis were among the least common findings, each noted in fewer cases (Table 6).

**Table 6:** MRI findings in study participants with bone involvement * Multiple responses for each study participant allowed

MRI findings*	Frequency (N = 105)	Percentage
Marrow edema	44	41.9%
Subchondral/intraosseous cyst	24	22.9%
Contusion	9	8.6%
Erosion	11	10.5%
Fracture	18	17.1%
Serpiginous area of altered signal intensity	2	1.9%
Subchondral compression	1	1%
Stieda process	8	7.6%
Sclerotic lesion	1	1%
Intraosseous lipoma	1	1%
Neoplastic lesion	3	2.9%
Osteophyte	8	7.6%
Periosteal reaction/periostitis	3	2.9%
Sequestrum	2	1.9%
Abscess	1	1%
Apophysitis	1	1%

We further analyzed the joint involvement among the participants (Table 7). The tibiotalar joint was the most frequently affected, with abnormalities observed in 76.2% (80 cases) of the participants, followed by the subtalar joint (talocalcaneal), with 58.1% (61 cases) of participants showing abnormalities. Other joints showed less frequent involvement. The distal tibiofibular joint was affected in 20% (21 cases) of participants, reflecting its role in ankle syndesmosis injuries. The talonavicular joint had a 9.5% (10 cases) involvement rate, which is important for the flexibility and movement of the midfoot. Less common joint involvements included the calcaneonavicular joint (5.7%, or six cases), the calcaneocuboid joint (4.8%, or five cases), the cuneonavicular joint (4.8%, or five cases), and the tarsometatarsal joint (3.8%, or four cases).

**Table 7:** Joint involvement among study participants * Multiple responses for each study participant allowed

Joint*	Frequency (N = 105)	Percentage
Tibiotalar joint	80	76.2%
Subtalar joint (talocalcaneal)	61	58.1%
Tarsometatarsal joint	4	3.8%
Distal tibiofibular joint	21	20%
Talonavicular	10	9.5%
Calcaneocuboid	5	4.8%
Cuneonavicular	5	4.8%
Calcaneonavicular	6	5.7%

The MRI findings in the patients with joint involvement showed that effusion was the most prevalent finding, present in 86 cases, or 81.9% of the sample. Reduced joint space was observed in 12 cases (11.4%). Synovial thickening was noted in 14 cases (13.3%), while subchondral erosions and cartilage loss were found in 10 (9.5%) and nine (8.6%) cases, respectively. Dislocations and loose bodies each appeared in four cases (3.8%). Periarticular collections were seen in three cases (2.9%), and synovial cysts were the least common, present in just one case (1%) (Table 8).

**Table 8:** MRI findings in study participants with joint involvement * Multiple responses for each study participant allowed

MRI findings*	Frequency (N = 105)	Percentage
Effusion	86	81.9%
Reduced joint space	12	11.4%
Dislocation	4	3.8%
Subchondral erosions	10	9.5%
Synovial thickening	14	13.3%
Periarticular collection	3	2.9%
Synovial cyst	1	1%
Cartilage loss	9	8.6%
Loose bodies	4	3.8%

We also studied the tendons involved. The Achilles tendon was the most frequently observed, with 41 cases, representing 39% of the total. The tibialis posterior tendon followed closely, with 45 cases, accounting for 42.9%. Flexor tendons were also notably prevalent, appearing in 40 cases, or 38.1% of the study. In contrast, the tibialis anterior tendon was the least frequently observed, with only four cases, making up just 3.8%. The extensor tendons were seen in 11 cases, equating to 10.5%, while the peroneus tendons were present in 34 cases, which is 32.4% of the total (Table 9).

**Table 9:** Tendon involvement among study participants * Multiple responses for each study participant allowed

Tendon*	Frequency (N = 105)	Percentage
Achilles	41	39%
Tibialis anterior	4	3.8%
Tibialis posterior	45	42.9%
Flexor	40	38.1%
Extensor	11	10.5%
Peroneus	34	32.4%

The MRI findings in study participants with tendon involvement showed that peritendonitis or tenosynovitis was the most frequently observed condition, present in 58 cases, which accounts for 55.2% of the study population. Tendinosis was also relatively common, found in 25 cases (23.8%). Partial tears were noted in 12 cases (11.4%), while complete tears were less frequent, occurring in seven cases (6.7%). Hematomas, ganglion cysts, abscesses, and encased tendons were among the least common findings, each observed in one to three cases, representing between 1% and 2.9% of the total (Table 10).

**Table 10:** MRI findings in study participants with tendon involvement * Multiple responses for each study participant allowed

MRI findings*	Frequency (N = 105)	Percentage
Peritendonitis/tenosynovitis	58	55.2%
Complete tear	7	6.7%
Partial tear	12	11.4%
Tendinosis	25	23.8%
Hematoma	1	1%
Ganglion cyst	3	2.9%
Abscess	1	1%
Encased	1	1%

The CFL was the most frequently involved, seen in 91 cases, which constitutes 86.7% of the study sample. The PTFL and ATFL were also commonly injured, with 39 (37.1%) and 37 (35.2%) cases, respectively. Involvement of the deltoid complex was seen in 15 cases, representing 14.3% of the sample. Pathologies of the posterior inferior tibiofibular ligament and the spring (calcaneonavicular) ligament were observed in 10 (9.5%) and seven (6.7%) cases each, respectively. The anterior inferior tibiofibular ligament was involved in seven cases (6.7%). Involvement of other ligaments, including the tibiocalcaneal, dorsal talonavicular, calcaneofibular, dorsal calcaneocuboid, and tibionavicular, was seen in very few cases, ranging from one to three cases each, indicating their relatively lower prevalence in the study (Table 11). The most frequently identified finding in the patients with ligament involvement was sprain, observed in 49 cases, which accounts for 46.7% of the study population. Partial tears were noted in 19 cases (18.1%), while complete tears were found in eight cases (7.6%). This distribution highlights sprains as the predominant type of ligament injury among the cases studied, with partial and complete tears being less common.

**Table 11:** Ligament involvement among study participants * Multiple responses for each study participant allowed

Ligament*	Frequency (N = 105)	Percentage
Anterior talofibular ligament	37	35.2%
Posterior talofibular ligament	39	37.1%
Calcaneofibular ligament	91	86.7%
Spring (calcaneonavicular ligament)	7	6.7%
Deltoid complex	15	14.3%
Posterior inferior tibiofibular ligament	10	9.5%
Anterior inferior tibiofibular ligament	7	6.7%
Tibiocalcaneal	3	2.9%
Dorsal talonavicular	1	1%
Calcaneofibular	1	1%
Dorsal calcaneocuboid	1	1%
Tibionavicular	1	1%

The involvement of the soft tissue was also analyzed in this study (Table 12). Subcutaneous tissue and skin were the most commonly affected, each observed in 79 cases, representing 75.2% of the sample. The retrocalcaneal bursa and Kager fat pad were also frequently involved, appearing in 26 (24.8%) and 16 (15.2%) cases, respectively. The calcaneal spur was seen in 18 cases (17.1%). In contrast, other soft tissues had much lower prevalence rates: the plantar fascia was involved in eight cases (7.6%), and the flexor hallucis longus muscle in nine cases (8.6%). Muscles such as the tibialis posterior, peroneus brevis, flexor hallucis brevis, extensor hallucis longus, and flexor digitorum longus were affected in one to five cases each, representing between 1% and 4.8%, indicating less frequent involvement.

**Table 12:** Soft tissue involvement among study participants * Multiple responses for each study participant allowed

Soft tissue involvement*	Frequency (N = 105)	Percentage
Subcutaneous tissue	79	75.2%
Skin	79	75.2%
Plantar fascia	8	7.6%
Tibialis posterior	1	1%
Peroneus brevis muscle	2	1.9%
Flexor hallucis brevis muscle	1	1%
Flexor hallucis longus muscle	9	8.6%
Extensor hallucis longus muscle	1	1%
Extensor digitorum longus muscle	5	4.8%
Flexor digitorum longus muscle	3	2.9%
Flexor digitorum brevis muscle	2	1.9%
Retrocalcaneal bursa	26	24.8%
Kager fat pad	16	15.2%
Sinus tarsi	3	2.9%
Calcaneal spur	18	17.1%

The MRI findings in participants with soft tissue involvement (Table 13) reveal that edema was the most common finding, present in 80 cases, which constitutes 76.2% of those with soft tissue issues. Bursitis was the next most frequent, identified in 26 cases, or 24.8%. Other notable findings included ulceration in nine cases (8.6%) and thickening in 11 cases (10.5%). Both abscesses and myositis/muscle edema were observed in seven cases each, representing 6.7% of the participants. Neoplastic lesions were found in three cases (2.9%), while muscle strain was the least common finding, seen in just one case (1.0%). Notably, there were no instances of foreign bodies or cystic lesions.

**Table 13:** MRI findings in study participants with soft-tissue involvement * Multiple responses for each study participant allowed

MRI findings*	Frequency (N = 105)	Percentage
Subcutaneous edema	80	76.2%
Bursitis	26	24.8%
Ulceration	9	8.6%
Skin thickening	11	10.5%
Abscess	7	6.7%
Myositis	7	6.7%
Neoplastic lesion	3	2.9%
Muscle strain	1	1.0%

## Discussion

Demography

In the present study, males accounted for 54% of the total study participants. The mean age of study participants was 39 ± 18.7 years. Participants aged 21 to 30 years made up the bulk of the study's sample.

In 2021, Turky et al. conducted an observational study among 60 patients to assess the function of MRI in identifying abnormalities related to post-traumatic ankle joint pain. A similar gender distribution was observed in the present study compared to the previous one. The mean age of participants was slightly lower (34.6 ± 15.95 years) [14]. However, the previous study included only patients with traumatic ankle joint pathology, while the present study included non-traumatic pathologies as well.

In 2022, Idowu et al. conducted a study to ascertain the prevalence, pattern, and range of abnormalities observed on ankle MRIs in adult Nigerians. A total of 50 adults were included; among them, 27 were males (54%) and 23 were females (46%). The age range of the participants was between 25 and 66 years, with a mean age of 42.84 ± 9.63 years [9]. The mean age was higher compared to the present study. Only adults above 25 years of age were selected in the previous study, while in the present study, age was not restricted.

Clinical characteristics

Over 90% of study participants presented with primary complaints of pain, of which two-thirds had a history of trauma. Around 68% of patients had a chronic history of illness (more than four weeks). About 76% of all the study participants had a history of trauma prior to the appearance of symptoms. Among the non-traumatic lesions (29 patients), 48% were due to inflammatory etiology, 34% could be attributed to infective causes, and the remaining 17% were neoplastic. The majority of the osseous lesions were found in the talus (39%), followed by the calcaneum (29.5%), lower tibia (21%), and lower fibula (15%). Over 75% of the study participants had lesions in the tibiotalar joint, 58% in the subtalar joint, and 20% in the distal tibiofibular joint. The tibialis posterior tendon (43%) was the most commonly involved, followed by the Achilles tendon (39%). Subcutaneous tissue involvement was seen in 75% of patients, primarily due to edema.

In Turky et al.'s study, 17 participants (28.3%) complained of acute onset of pain, while 43 participants (71.7%) had chronic onset of pain [14]. The present study showed similar findings, with 32 study participants (30.5%) complaining of acute onset of pain, while 67 cases (63.8%) were of chronic onset of pain.

In Idowu et al.'s study, 40 cases (80%) had a history of trauma, and 50% of the patients had ankle joint effusion [9]. Joint effusion was more frequently observed in the present study, seen in approximately 82% of patients. The proportion of trauma is higher than in similar studies conducted in India (38-80%) [15,16].

Outcomes

In the present study, the most frequently encountered MRI finding in the bones was marrow edema (41.9%), followed by subchondral/intraosseous cysts (22.9%), fractures (17.1%), cortical erosions (10.5%), and contusions (8.6%). MRI evaluation of the joint space revealed joint effusion in approximately 81.9% of cases, reduction in joint space in 11.4% of cases, synovial thickening in 13.3% of cases, and peri-articular cartilage loss in 9% of cases. The most frequently encountered tendon pathologies were peritendonitis/tenosynovitis (55.2%) and tendinosis (24%). Approximately 11.4% of the study participants had a complete tear of the tendon. Soft tissue involvement was excellently assessed in the MRI study and showed findings such as subcutaneous edema (76%), bursitis (25%), thickening of the plantar fascia (10%), and muscle edema (7%). MRI was also helpful in assessing ligamentous injuries and classifying them into sprain (46.7%), partial tear (18%), and complete tear (7.6%).

In the study done by Idowu et al., 50% of the cases had joint effusion, 8% had tendinosis of the Achilles tendon, 2% had tendinosis of the tibialis posterior tendon, and 12% had subcutaneous edema [9]. However, in the present study, 76% of the participants showed subcutaneous edema, and 82% showed joint effusion on MRI, which is significantly higher compared to the previous study. The probable reason could be the difference in demographics between the two studies. The higher proportion of joint effusion is likely due to the inclusion of more infectious and inflammatory pathologies in the present study compared to the previous study. Pain is a common presentation, documented in 90% of the study participants.

In the present study, 14% had deltoid complex pathology on MRI. This finding was significantly higher than in Idowu et al.'s study (8%) [9]. Significant force is needed to damage the deltoid ligament. In the present study, 18% had partial tears, and 7.6% had complete tears. The total proportion of tears is comparable between both studies.

Among the study participants in Turky et al.'s study, nine (16%) had ligament injuries, 29 (48.3%) had tendinous injuries, and 35 (58.3%) had osseous injuries. Out of the total, nine cases (15%) were diagnosed as fractures, one case (1.7%) was identified as bone necrosis, and 34 cases (56.7%) were determined to be bone contusions [14]. In the present study, bone involvement was seen on MRI in the form of marrow edema (41.9%), intraosseous cysts (22.9%), fractures (17.1%), cortical erosions (10.5%), and contusions (8.6%). Although the prevalence of fractures across both studies was comparable, the prevalence of contusions observed on MRI in Turky et al.'s study was significantly higher. Nearly 55 study participants (91.7%) exhibited joint effusion, while 11 (18.3%) showed symptoms of sinus tarsi syndrome in the previous study [14]. In the current study, 81.9% of the study participants showed joint effusion, and only four cases (3.8%) showed findings of sinus tarsi syndrome. The higher proportion of contusions, joint effusion, and sinus tarsi syndrome in Turky et al.'s study is probably due to the inclusion of only patients with post-traumatic ankle joint pathology.

Sayed et al. studied 50 patients with severe ankle pain in 2021. Among them, 28% were found to have ligament tears, with the ATFL being the most frequently torn. The study also observed Achilles pathology (14%), retrocalcaneal bursitis (4%), and various osseous lesions in 54% of patients. Bone marrow edema was observed in 32% of cases [17]. The present study revealed that 35.2% of cases had an ATFL, 25% had bursitis, and 41.9% had marrow edema. The prevalence of deltoid pathology observed in the previous study (12%) was similar to the findings of the current study.

In 2021, Bajwa et al. conducted an observational study among 61 participants, with the majority in the younger age group of 21-30 years (27.87%). Half of them had trauma. Twelve participants exhibited MRI evidence of infection, while five revealed neoplastic pathologies [18]. In the present study, among the non-traumatic lesions, 48% were due to inflammatory etiology, 34% were attributed to infective causes, and the remaining 17% were neoplastic. The prevalence of neoplastic and inflammatory etiologies in the present study was higher than in the Bajwa et al. study.

Although the present study contributes valuable insights to the field, it has several limitations. The relatively small sample size may not be representative of broader populations. Additionally, the study lacks long-term follow-up data on the progression of the pathology, its treatment, and outcomes. The inclusion of both traumatic and non-traumatic lesions may complicate the findings, and potential confounding factors, such as pre-existing conditions, could not be fully controlled.

## Conclusions

MRI excels in diagnosing ankle and hindfoot pathologies due to its superior soft tissue contrast, surpassing conventional radiography and CT in detecting tendon, ligament, and osseous abnormalities. It effectively identifies conditions such as osteomyelitis, plantar fasciitis, and neoplasms, which are generally benign. MRI is crucial for evaluating injuries, particularly to the lateral ankle ligaments and Achilles tendon, and is essential for diagnosing trauma-related osteoarthritis. This study highlights the frequent involvement of the talus and calcaneum, with the tibiotalar and subtalar joints being the most affected. Future research should focus on correlating MRI findings with intraoperative observations to assess diagnostic accuracy.
